# Supplementary material for: The Orphan GPR50 Receptor Regulates the Aggressiveness of Breast Cancer Stem-like Cells via Targeting the NF-kB Signaling Pathway
Source: Int J Mol Sci. 2023 Feb 1;24(3):2804. doi: 10.3390/ijms24032804 (PMC9917945; doi:10.3390/ijms24032804)
Supplement: Supplementary file 1 [file ijms-24-02804-s001.zip › ijms-2150338-supplementary.pdf]

Supplementary Data S1

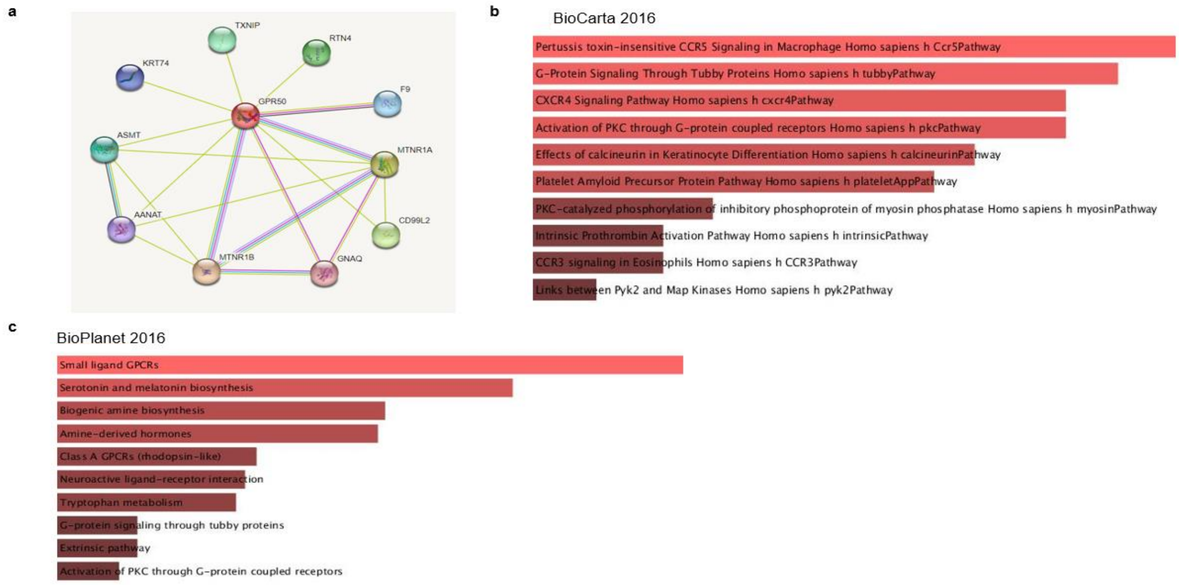

Figure S1. Interacting protein profile with the GPR50 involved in signaling pathways in Breast Cancer. (a) PPI profile of GPR50 was analyzed using STRING (v. 11.5). Enrichr Bar Graph data were collected from Enricher web (https://amp.pharm.mssm.edu/Enrichr).
(b) BioCarta pathways 2016. (**c**) BioPlanet pathways 2016.
